# Sequence Analysis and FISH Mapping of Four Satellite DNA Families among Cervidae

**DOI:** 10.3390/genes11050584

**Published:** 2020-05-24

**Authors:** Miluse Vozdova, Svatava Kubickova, Halina Cernohorska, Jan Fröhlich, Natália Martínková, Jiri Rubes

**Affiliations:** 1 Department of Genetics and Reproduction, Central European Institute of Technology—Veterinary Research Institute, Hudcova 70, 621 00 Brno, Czech Republic; kubickova@vri.cz (S.K.); cernohorska@vri.cz (H.C.); frohlich@vri.cz (J.F.); rubes@vri.cz (J.R.); 2 Institute of Vertebrate Biology, Czech Academy of Sciences, Kvetna 8, 603 65 Brno, Czech Republic; martinkova@ivb.cz

**Keywords:** centromere, cervid phylogeny, FISH, satellite DNA, sequencing

## Abstract

Centromeric and pericentromeric chromosome regions are occupied by satellite DNA. Satellite DNAs play essential roles in chromosome segregation, and, thanks to their extensive sequence variability, to some extent, they can also be used as phylogenetic markers. In this paper, we isolated and sequenced satellite DNA I-IV in 11 species of Cervidae. The obtained satellite DNA sequences and their chromosomal distribution were compared among the analysed representatives of cervid subfamilies Cervinae and Capreolinae. Only satI and satII sequences are probably present in all analysed species with high abundance. On the other hand, fluorescence in situ hybridisation (FISH) with satIII and satIV probes showed signals only in a part of the analysed species, indicating interspecies copy number variations. Several indices, including FISH patterns, the high guanine and cytosine (GC) content, and the presence of centromere protein B (CENP-B) binding motif, suggest that the satII DNA may represent the most important satellite DNA family that might be involved in the centromeric function in Cervidae. The absence or low intensity of satellite DNA FISH signals on biarmed chromosomes probably reflects the evolutionary reduction of heterochromatin following the formation of chromosome fusions. The phylogenetic trees constructed on the basis of the satellite I-IV DNA relationships generally support the present cervid taxonomy.

## 1. Introduction

The Cervidae (Ruminantia, Mammalia) family includes more than fifty different species divided into three subfamilies: Cervinae, Capreolinae, and Hydropotinae [1]. Two tribes (Cervini and Muntiacini) are recognised among Cervinae [2]. Several of the Cervidae species have a growing economic potential as farm animals, but most of the species within the group are endangered and need international protection. The diploid chromosome number ranges from 2n = 70 in Capreolinae and Hydropotinae and up to 2n = 6 in the Indian muntjac (*Muntiacus muntjak vaginalis*) female [3,4,5]. Chromosomal evolution in Cervidae is largely attributable to Robertsonian fusions, although muntjacs (Muntiacini) are known for tandem fusions reducing their diploid numbers from 2n = 46 in Chinese muntjac (*Muntiacus reevesi*) to 2n = 6/7 in *M*. *vaginalis* [6,7,8].

It is known that a significant proportion of eukaryotic genomes consists of constitutive heterochromatin, a genomic fraction that includes satellite DNAs, short interspersed nuclear elements (SINEs), long interspersed nuclear elements (LINEs), and other repetitive elements. Among them, satellite sequences are one of the most dynamic parts of higher eukaryotic genomes. Being present in large quantities, the tandemly repeated satellite DNAs can account for up to 10–20% of mammalian genomes [9]. It is not uncommon that several satellite families are found clustered together in certain chromosomal regions, most often around the centromeres [10,11], contributing thus to the complex structure of peri/centromeric DNA [12]. Many satellite sequences and their overlying heterochromatin were recognised to have essential roles tied to chromosome segregation [13] and, therefore, are being subject of many current investigations in diverse mammals.

New satellite sequences could be formed de novo [14] or originate from pre-existing satellite DNAs [15,16], and can coexist with the progenitor satellite DNAs [17]. Closely related species share similar satellite sequences [18], and thus, the satellite DNA can be used as a phylogenetic marker in evolutionary studies [19,20,21,22,23,24,25,26]. The differences in satellite sequences among taxa can, to some extent, be used for approximation of the species divergence times [27]. The sequence similarity of satellite DNA family can group species with a monophyletic origin [18,24,26,28,29]. In Cervidae, four different centromeric satellite DNA families were previously detected, with satI and satII DNA being the most common [30,31,32]. In the genus Muntiacus, also, satV and satVI families have been described [32]; nevertheless, their presences in other cervid species have not been confirmed. Moreover, most of the investigations referred above focused on the satellite DNA sequences but not on their chromosomal localisation, especially in deer species other than Muntiacus. Therefore, the aim of our study was to isolate various types of cervid satellite DNAs, analyse their sequences, determine their chromosome positions by fluorescence in situ hybridisation (FISH), compare their hybridisation patterns in cross-species FISH, and use the obtained data for a construction of a phylogenetic tree reflecting satellite DNA relationships among the analysed 11 species from subfamilies Cervinae and Capreolinae.

## 2. Material and Methods

### 2.1. Samples

Samples of whole peripheral blood of seven Cervinae: the red deer (*Cervus elaphus*, CEL, 2n = 68), milu deer (*Elaphurus davidianus*, EDA, 2n = 68), rusa deer (*Rusa timorensis*, RTI, 2n = 60), Eld’s deer (*Rucervus eldii*, REL, 2n = 58), fallow deer (*Dama dama*, DDA, 2n = 68), white-lipped deer (*Cervus albirostris*, CAL, 2n = 66), and Chinese muntjac (*Muntiacus reevesi*, MRE, 2n = 46); four Capreolinae: moose (*Alces alces*, AAL, 2n = 68), reindeer (*Rangifer tarandus*, RTA, 2n = 70), roe deer (*Capreolus capreolus*, CCA, 2n = 70), and white-tailed deer (*Odocoileus virginianus*, OVI, 2n = 70); and two outgroup species of Bovidae: cattle (*Bos taurus*, BTA, 2n = 60) and sheep (*Ovis aries*, 2n = 54) were obtained from captive-born animals held in the Czech zoological gardens in Prague and Dvur Kralove and/or on private deer farms in Bila Lhota and Frycovice, Czech Republic. Samples were cultivated, harvested, and fixed according to the previously described protocol [33] and used for the preparation of metaphase chromosome spreads for FISH analysis.

All procedures performed in this study were in accordance with the ethical standards of the Veterinary Research Institute (Brno, Czech Republic), which complies with the Czech and European Union Legislation for the protection of animals used for scientific purposes. According to these regulations, ethics approval was not required, as the biological material (blood/tissue) was obtained post-mortem from animals upon animal slaughter in abattoir or which died during hunting. The blood from living animals was collected by a zoo veterinarian during other medical procedures. All collaborating zoos have licenses issued by the Ministry of the Environment of the Czech Republic (Act No 162/2003 Coll.).

### 2.2. Satellite DNA Isolation

Blood samples (200 µL) were used for genomic DNA isolation using the QIAamp DNA Blood Mini Kit (Qiagen, Hilden, Germany). SatI-IV DNA sequences were isolated from total genomic DNA by PCR amplification using primers designed according to the published NCBI (National Center for Biotechnology Information) sequences. All PCR reactions were performed using a Hot Start Combi PPP Master Mix (Top-Bio, Prague, Czech Republic) according to the manufacturer’s instructions. The NCBI accession numbers of the original sequences, designed primers, and PCR product lengths are displayed in Table 1. PCR products were subsequently cloned into the pDrive Cloning Vector (Qiagen, Hilden, Germany). In each species, four different clones of satI and satII and two clones of satIV were selected and subjected to sequencing. SatIII DNA sequences were obtained only in the four Capreolini species, and one clone per species was sequenced. PCR with satIII partial primers (Table 1) was used to confirm the presence of the satIII DNA in the remaining cervid species.

### 2.3. Sequence Analysis

The satI-IV sequences obtained in this study were screened for interspersed repeats by RepeatMasker (http://www.repeatmasker.org), and their GC content was calculated using the DNA/RNA GC Content Calculator (http://www.endmemo.com). All satellite sequences were screened for the presence of a 17-bp CENP-B-binding motif NTTCGNNNNANNCGGGN using FIMO (version 5.1.0) software (http://meme-suite.org) [34]. The FIMO software was also used for a search for the 31-bp subrepeat unit motif [35] in our satI DNA sequences. The sequences obtained in this study were compared to cervid satellite sequences available in the NCBI database using BLASTN (version 2.10.0) (https://blast.ncbi.nlm.nih.gov). BLAST2 software was used to assess the sequence homology.

### 2.4. Phylogenetic Analysis of the Satellite DNAs

The outgroup satellite DNA sequences did not align unambiguously with cervid sequences, and thus, the phylogenetic analysis was performed on the ingroup only. Multiple sequence alignments were constructed in MAFFT 7.4 [36] using the E-INS-I algorithm [37] for each satellite sequence separately. Optimal substitution models were selected with the smart model selection algorithm in PhyML [38,39] based on the Bayesian information criterion. Prior to the phylogenetic analysis, the indels in the alignments were recoded to the presence/absence of data to capture phylogenetic information in the indels [24]. The phylogenetic trees were reconstructed in MrBayes 3.2 [40] in a partitioned analysis, capturing the DNA sequence variation and the phylogenetic information in the indels. The Markov Chains Monte Carlo (MCMC) were run for 2 mil. (satI, satII, and satIV) or 0.5 mil. generations (satIII), sampled every thousandth generation to ascertain MCMC convergence at 30% burnin. The trees were rooted and visualised in phytools 0.6 [41,42], where nodes with posterior probability ≥0.95 were considered supported.

### 2.5. Fluorescence in Situ Hybridisation

Probes for satI, satII, satIII-partial, and satIV of *C. elaphus* (Cervinae) and satI-IV of *R. tarandus* (Capreolinae) were labelled with orange—or green—dUTP and used for comparative FISH. Moreover, we used satI (NCBI accession numbers: V00124 and Z18540) and satII (NCBI accession numbers: M36668 and AF245169) probes derived from two bovid species (*B. taurus* and *O. aries*). The FISH was carried out according to standard protocols [26]. Hybridisation signals were examined using an Olympus BX60 fluorescence microscope equipped with appropriate fluorescent filters. Images of well-spread metaphase cells were captured by a CoolCube CCD camera and analysed using ISIS3 software (version 5.8.3, MetaSystems, Altlussheim, Germany).

## 3. Results

### 3.1. Sequence Analysis

SatI, satII, and satIV DNAs were isolated from the seven species of Cervinae, and satI-IV were analysed in the four species of Capreolinae. SatIII sequences were isolated only from Capreolinae, because no PCR amplification product was obtained using satIII specific primers in any of the Cervinae species. However, using primers specific to the partial satIII sequence, we obtained PCR products of 578 bp in all Cervidae. All obtained satellite DNA sequences were deposited in the NCBI database under accession numbers MT185959-MT186072. The PCR product lengths, GC content, and sequence similarities of the clones are displayed in Table 2.

Our search for the 31-subrepeat unit of satI DNA revealed this motif in all analysed sequences, but its copy numbers varied in the studied species (Table 2). The analyses of satI, satII, and satIV sequences by RepeatMasker did not reveal any SINE, LINE, or LTR elements. In contrast, satIII DNAs showed the presence of L1 and retrotransposable elements (RTE) occupying approximately 20–23% of the satIII sequence.

Pairwise sequence comparisons of the individual satellite DNA families within and among the cervid tribes or subfamilies are displayed in Table 3. None of the cervid satellite DNA sequences shared any similarity with satellite DNAs from *B. taurus* and *O. aries* available in the NCBI database.

The screening of all satI-IV sequences for the CENP-B-binding motif revealed its presence in satII DNA of all analysed species except *A. alces* (Figure S1). The positions of the predicted CENP-B-binding motif in the satII DNA sequence varied among the individual cervid tribes: It was detected in positions 108–124 of the satII DNA sequence in M. reevesi, in positions 146–162 in Capreolinae, and in positions 222–238 in Cervini.

### 3.2. Fluorescence in Situ Hybridisation

**Figure 1:**
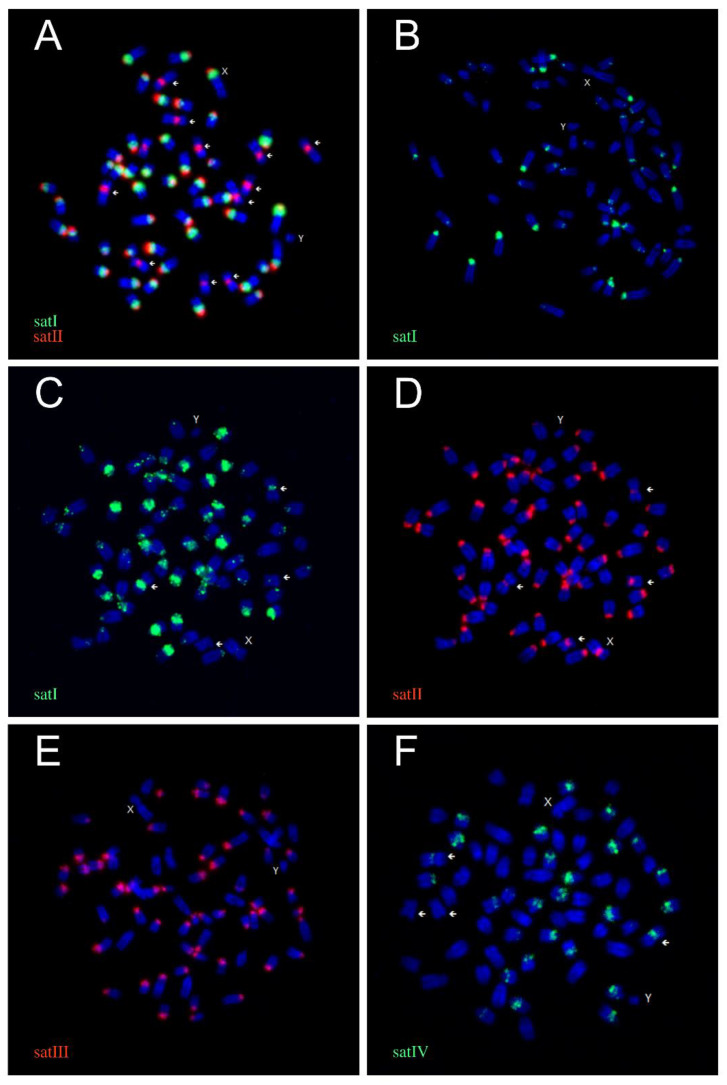
Examples of the fluorescence in situ hybridisation (FISH) patterns of satI-IV probes in Cervidae. (**A**) SatI (green) and satII (red) probes in *Rucervus eldii* (Cervini). (**B**) SatI (green) probe in *Capreolus capreolus* (Capreolini). (**C**) SatI (green) probe in *Alces alces* (Capreolini). (**D**) SatII (red) probe in *A. alces*. (**E**) SatIII probe (green) in *C. capreolus*. (**F**) SatIV probe (green) in *A. alces*. Arrows indicate metaphase chromosomes.

We used satI, satII, and satIV sequences isolated from *C. elaphus* for FISH in the seven Cervinae species. The satI-IV probes originating from *R. tarandus* were hybridised in the four species of Capreolinae. The results are summarised in Table 4 and Figures S2–S5, and examples of the observed FISH patterns are demonstrated in Figure 1. An example of *R. eldii* karyotype after FISH with the satI and satII probes is displayed in Figure S6.

The satI and satII probes produced peri/centromeric FISH signals in all analysed cervid species. Signals of the satII probe were detected in the centromeric regions of all autosomes and the X chromosomes.

The satI probe signals were located distally to the satII domain or partially overlapped it. The satI signals were mostly limited to the acrocentric chromosomes, including the acrocentric X. The biarmed chromosomes showed satI signals only in *A. alces* and *O. virginianus*. Only part of the chromosomes was labelled by the satI probe in *C. capreolus* and *A. alces*.

Regarding the satIII probe, no FISH signals were detected in Cervinae. In contrast, the satIII probe hybridised to centromeres of the majority of acrocentric autosomes in Capreolini, with the exception of *A. alces* lacking satIII signals. Biarmed chromosomes showed satIII signals (weak) only in *O. virginianus*.

SatIV signals were observed on a part of the autosomes in *M. reevesi*, *A. alces*, and *O. virginianus*. No signals of the satIV probe were detected in Cervini, *C. capreolus*, and *R. tarandus*.

None of the satI-IV probes produced FISH signals on the Y chromosome in any of the species.

### 3.3. Outgroup FISH with satI and satII DNA in Bovidae and Cervidae

The cross-hybridisation results are displayed in Figure S7. The *C. elaphus* satI probe hybridised to centromeric regions of most autosomes of *B. taurus* (Bovinae, 2n = 60; all autosomes are acrocentric). In *O. aries* (Antilopinae, 2n = 54), the cervid satI probe hybridised to centromeres of all acrocentric autosomes, while the three biarmed chromosomes (BTA1;3, BTA2;8, and BTA5;11 orthologs) lacked signals. In contrast, satI probes derived from *B. taurus* and *O. aries*, respectively, failed to show any hybridisation signals in cervid species representing Cervinae and Capreolinae. In autologous FISH, the satI probe derived from *B. taurus* showed signals to all *B. taurus* autosomes. The satI probe derived from *O. aries* showed fluorescence to all *O. aries* acrocentric autosomes, while weak signals were observed on the BTA2;8 and BTA5;11 orthologs, and BTA1;3 was unpainted. No signals were observed when the *B. taurus* satI probe was used to *O. aries* chromosomes and vice versa.

The *C. elaphus* satII probe hybridised neither to *B. taurus* nor to *O. aries*. Similarly, no signals were observed when satII probes derived from *B. taurus* and *O. aries* were used to the *E. davidianus* and *R. tarandus*, respectively. In autologous FISH, the bovine and ovine satII probes showed weak hybridisation to all autosomes in both species. Similarly, weak signals were observed in FISH experiments using the *B. taurus* satII probe to *O. aries* chromosomes and vice versa. Sex chromosomes were unpainted in all cases.

### 3.4. Phylogenetic Analysis of the Satellite DNAs

**Figure 2:**
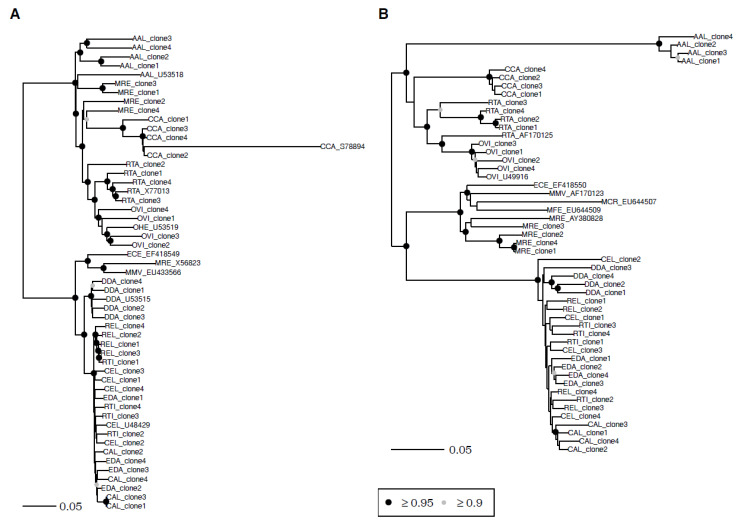
Bayesian phylogenetic trees constructed from cervid satellite sequences. (**A**) SatI and (**B**) satII. AAL—*Alces alces*, CAL—*Cervus albirostris*, CCA—*Capreolus capreolus*, CEL—*Cervus elaphus*, DDA—*Dama dama*, ECE—*Elaphodus cephalophus*, EDA—*Elaphurus davidianus*, MCR—*Muntiacus crinifrons*, MFE—*Muntiacus feae*, MMV—*Muntiacus muntjak vaginalis*, MRE—*Muntiacus reevesi*, OHE—*Odocoileus hemionus*, OVI—*Odocoileus virginianus*, REL—*Rucervus eldii*, RTA—*Rangifer tarandus*, and RTI—*Rusa timorensis*. Circles at nodes signify nodes with posterior probability ≥0.95 (black) and ≥0.90 (grey). Unmarked nodes were not supported.

Together with sequences previously published in the NCBI database, multiple sequence alignments consisted of 7–53 satI-IV sequences of species from the family Cervidae (Table 5). In the satI phylogeny, the satellite sequences corresponded to current taxonomy at the subfamily and tribe levels, with the exception of the tribe Muntiacini (Figure 2A). Sequences of clones 1-4 isolated from *M. reevesi* formed an unsupported paraphyletic relationship with satI from Alcini and Capreolini. This pattern was not observed in the satII phylogeny (Figure 2B). In the satII phylogeny, Capreolinae included significantly supported tribes Alcini, Capreolini, and Rangiferini. In Cervinae, the satII sequences were distinct between Muntiacini and Cervini. Phylogenies of satIII and satIV depicted shallow and often unsupported relationships at all included taxonomic levels (Figure S8).

## 4. Discussion

In Cervidae, six satellite DNA families have been identified to date, of which satV and VI were detected in the genus *Muntiacus* [30,31,32]. The vast majority of sat DNA sequences so far deposited in the NCBI database were obtained by density-gradient centrifugation. In our approach, specific sets of primers were designed to amplify four major groups of satellite DNA (satI-IV) in cervid species. The resulting sequences were subjected to comparative studies within Cervidae, including both sequence comparisons and physical localisation by FISH on metaphase chromosomes.

Our comparative sequence analysis revealed relatively high sequence similarity among the individual sat DNA families and cervid tribes. Particularly high satI, satII, and satIV DNA similarities were observed among Cervini, indicating close relationships within this clade. Interestingly, the satI and satII sequences of Muntiacini, a sister clade to Cervini, were more similar in their lengths to Capreolinae than to Cervini (Table 2). Although not reflected at the level of their sequence similarity (Table 3), satI and satII lengths similarity between Muntiacini and Cervini manifested as *M. reevesi* grouping within Capreolinae in the satI phylogeny and within Muntiacini in satII (Figure 2). Nevertheless, we must not forget that only one species representative of Muntiacini (*M. reevesi*) was available for this study. Previously published sequences of Muntiacini satI were included in the Cervinae subfamily, as expected (Figure 2A, accession numbers X56823 and EU433566) [43,44].

### 4.1. SatI DNA

The centromeric satI DNA is the most common satellite family found not only throughout all deer species but, also, in a wide range of Bovidae and Antilocapridae (Ruminantia) [12,24,45,46,47]. It was postulated that both cervid and bovid satI arose from an initial subrepeat unit of a 31-bp DNA sequence [12,31,35]. In deer, the 31-bp subrepeat unit is organised into monomers that differ in size between two paleontologically recognised groups [48]: as a 0.8-kb monomer in plesiometacarpalia and a 1-kb monomer in telemetacarpalia [12]. We detected variable copy numbers of the 31-bp subrepeat unit in satI DNA of all cervid species analysed in this study. Despite this, no significant sequence similarity between cervid and bovid satI DNA was found. The satI monomer isolated from Cervini in this study was approximately 200 bp shorter than that isolated from *M. reevesi* (Muntiacini) and from Capreolinae (Table 2), which corresponds with previous studies [12].

In Ruminantia, satI DNA usually occupies 8–12% of the genomic content, but up to 35% of the total nuclear DNA content is formed by satI sequences in *A. alces* [49]. In this study, a high abundance of satI DNA was also detected by FISH in *R. eldii* and other cervid species (Figure 1). In contrast, about half of the acrocentric autosomes in *C. capreolus* showed only weak or missing satI signals, indicating that another satellite DNA probably predominates in *C. capreolus* centromeres. Notably, this could be similar to the previously published, divergent, and shorter satI sequence from *C. capreolus* (Figure 2A, accession number S78894) [46]. Strong signals of the satI probe were detected in centromeric regions of acrocentric autosomes, whereas the biarmed autosomes showed only weak or no signals. Similar hybridisation patterns were also observed for other satellite DNA probes in this study and were previously reported in numerous species of Ruminantia, particularly in bovids [28,49,50,51]. This can be explained by a gradual evolutionary reduction in centromeric heterochromatin on fused biarmed chromosomes [10,24,52]. The reduction in centromeric heterochromatin in evolutionarily fused biarmed chromosomes is considered to reflect the antiquity of the fusion event—the older the fused chromosome, the less centromeric heterochromatin is retained [24,28,51,53,54,55].

### 4.2. SatII DNA

SatII DNA sequences are less abundant than the satI DNA in most Cervidae and may represent 2–4% of the genomic DNA [49]. Originally, the cervid satII family was isolated from *O. virginianus* and characterised by monomeric repeats of approximately 0.7 kb with 67% GC content [56]. Later on, it was also described in the Indian muntjac (*M. muntjak*) and several other cervid species [11,21,57]. The absence of any internal repetition and the similar length of the satII DNA repeat units (700 bp repeated in tandem) described in Cervidae were also reported in Bovidae [56,58], indicating the conservativeness of the sequence. Our data support the satII sequence as conservative in Cervidae as evidenced by its good congruence with taxonomic divergences (Figure 2B). However, our comparison of the satII sequences derived from cervid clones isolated in this study with the corresponding bovid sequences archived in the NCBI database did not reveal any sequence similarities. It was previously postulated [59] that the evolution of satII DNA in Bovidae and Cervidae occurred mainly by base substitutions from an ancestral 700-bp tandem repeat, which could have resulted in the observed similarity loss.

Our FISH experiments with satI-IV DNA probes showed that only the satII probe hybridised to the centromeres of all (both acrocentric and biarmed) autosomes and X chromosomes in all analysed species of Cervinae and Capreolinae. Although there are no FISH data on Hydropotinae in this study, we assume that satII sequences may represent the most important satellite family that might be involved in the centromeric function in Cervidae. This hypothesis is supported by several facts:

(i) The satII probe signals were located more proximally compared with the pericentromeric satI probe motifs by double-colour FISH. Similar hybridisation patterns have already been described in *Hydropotes inermis* and *M. reevesi* [21,60].

(ii) FISH signals of the satII probe showed similar intensities on the acrocentric and biarmed autosomes and X chromosomes in all studied species.

(iii) Centromeres of the biarmed, evolutionarily young, rearranged BTA1 dist. orthologs in *A. alces* and *R. tarandus* [61] showed hybridisation with satII probes, while satI, satIII, and satIV probes produced only week or no signals on this chromosome.

(iv) A significantly higher GC content (60–68%), which is considered to be compatible with the centromeric function [62], was revealed in satII compared to satI, satIII, and satIV sequences (44–55%).

(v) The CENP-B motif was revealed in satII clones from all but one analysed cervid species. It was previously shown that cervid satII DNA serves as a target for binding of the CENP-A centromeric protein, which is believed to define the centromere identity [63,64]. The CENP-B protein is known to directly interact with CENP-A and a set of other proteins that form the constitutive centromere-associated network [65]. In this study, the CENP-B motif was not recognised only in *A. alces*, probably due to its more extensive sequence variation. Interestingly, *A. alces* also showed the lowest GC content of the satII DNA of all analysed cervid species.

### 4.3. Satellite III Sequences

The satIII DNA, with a repeat unit of 2.2 kb, was initially described in *C. capreolus*, and until its identification in the *H. inermis* genome, was believed to be specific to *C. capreolus* [14,60]. In this study, the 2-kbp satIII repetitive unit was successfully isolated from all Capreolinae. Moreover, a partial satIII repetitive sequence of approximately 580 bp was detected by PCR in all species studied here. This partial sequence might represent an ancestral satIII fragment that was retained in cervid genomes after evolutionary diversification of their satellite DNA sequences. FISH signals of the satIII probe derived from RTA chromosomes were observed only in *C. capreolus*, *R. tarandus*, and *O. virginianus*. However, in *C. capreolus*, the satIII probe produced the most intense signals of the four (satI-IV) satellite probes used. This is in accordance with the previous finding that satIII represents the prominent satellite DNA in *C. capreolus*, accounting for approximately 5–10% of its genome [31]. The lack of detectable FISH signals in most cervid species analysed in this study can be attributed to low satIII DNA copy numbers.

The presence of the L1 and RTE sequences were detected in the satIII DNA in Capreolini. A similar association of transposable elements (TE) with satellite DNA was previously reported in Bovidae [66]. TEs have been suggested to play a role in satellite DNA evolution, genomic expansion, and movements, and their presence might be associated with the centromeric activity [66,67,68,69].

### 4.4. Satellite IV Sequences

Cervid satIV DNA with a repeat unit of approximately 1 kb was originally isolated from *M. muntjak* and *M. reevesi*, but the sequence was subsequently also detected in several other cervid species [57,70]. SatIV repeats were also described in the family Bovidae [10], but no sequence homology with the cervid satIV DNA was detected in our study. We observed a high sequence conservation among the satIV DNA clones in the individual species, as well as among the species (>93%). The only exception was *C. capreolus*, whose satIV DNA showed only 85% similarity with satIV sequences of all other analysed species. Our finding of high satIV sequence similarities is in accordance with previous studies [57]. This might be associated with the functional centromeric role of this DNA, which was previously suggested from its co-localisation with centromeric proteins at the kinetochore [57]. The distribution of satIV sequences to all centromeres have previously been documented for representatives of the genus *Muntiacus* [57,70]. In our FISH experiments, signals of the satIV probe were detected on a part of the chromosomes of *M. reevesi*, *A. alces*, and *O. virginianus*. No FISH signals were observed in any other cervid species. Similar to the satIII DNA, the FISH results were probably limited by relatively low numbers of the satIV repeats in most of the analysed species.

### 4.5. Outgroup FISH Comparisons

The retention of satI-IV in all cervid species studied suggests that these sequences emerged before their separation to phylogenetic lineages. No outgroup similarity of cervid satI-IV DNA with bovid satellite DNA sequences available in the NCBI database was detected in this study. This can document the independent evolution of satellite DNAs in Cervidae and Bovidae after the clades’ separations.

Despite the fact that cervid satI and satII DNA share no similarity with bovid satI and satII sequences, the cervid satI probe produced FISH signals in the centromeric regions of bovine and ovine chromosomes. In contrast, no FISH signals were detected in Cervidae using satI and satII probes derived from cattle or sheep. The possible explanation of this reciprocal hybridisation failure could be based on the process of satellite DNA evolution. It is known that new satellite DNA variants are formed during speciation from pre-existing sequences and either replace them or coexist with the ancestral satellites [17,18,24]. Our results indicate that bovid centromeres probably cluster the ancestral variants of satI DNA retained also in Cervidae with new satI sequences developed in the evolution of modern bovids after their separation from Cervidae.

## 5. Conclusions

Our results showed that satII DNA probably represents the most important satellite DNA family in Cervidae. The phylogenetic analysis of the satellite DNA sequences produced data congruent with the current deer taxonomy.

## Figures and Tables

**Table 1:** Original sequences, primers, predicted amplicon sizes, and annealing temperatures.

	Original Sequence	Species of Origin	Primers for Amplification	Product Length (Predicted)	Annealing Temperature
SatI	U48429.1	*Cervus elaphus*	CAAGACGAAAGGATGTCTGAATCC	727 bp	58 ^o^C
			GGTGTCCATTCCACTTGAGGC		
SatII	U49916.1	*Odocoileus virginianus*	CTGTGAGTGTGGAGCCCCGAGC	580 bp	64 ^o^C
			GTGGGAAGAGGCAGAGCCGACC		
SatIII	Y10686.1	*Capreolus capreolus*	GAGGTGTGACCGTGACAGGACCC	2030 bp	64 ^o^C
			CGAGGCTGGGATGTTGTGAGAAGC		
SatIII-partial		*C. capreolus*	ACAGATGGAGAACATCCCTCTGG	578 bp	57 ^o^C
			GTGAATACGAAAAGGACTGTGGG		
SatIV	AY064469.1	*O. hemionus*	TTGATATTAGGTGATTGGATGGG	728 bp	57 ^o^C
			AAGATGTCAGAACTTCAGGTTTGC		

**Table 2:** Characteristics of the satI-IV sequences. The data are based on analyses of four clones of each satellite DNA per animal.

Species	SatI				SatII			SatIII		SatIV		
	Length (bp)	GC Content (%)	Similarity (%)	No. of 31-bp Units	Length (bp)	GC Content (%)	Similarity (%)	Length (bp)	GC Content (%)	Length (bp)	GC Content (%)	Similarity (%)
CEL	724–725	54	94–96	21	655–656	67	89–97			727–728	45	97
DDA	725–726	54	93–94	21	654–664	67	89–93			727	45	99
REL	723–725	54	96–99	20	655–657	68	95–98			727–728	46	98
CAL	724–725	54	94–100	20	655–656	68	94–97			726–727	45	97
EDA	723–725	55	93–96	20	656	68	96–98			727	46	100
RTI	725	55	94–96	20	654–656	67	93–97			726–727	46	97
MRE	904–908	51	81–93	27	588–590	63	86–99			727	44	99
CCA	909–910	48	88–98	21	578	63	96–98	2000	51	725	44	99
AAL	906–911	50	79–88	27	522–523	60	92–98	2025	52	723–724	45	96
RTA	906–911	53	82–93	26	573–577	65	87–99	2018	52	726–727	45	99
OVI	908–910	52	77–87	25	578–580	66	94–97	2073	52	726–727	45	96

**Table 3:** Satellite DNA sequence similarity among Cervini, Muntiacini, and Capreolinae.

		SatI			SatII			SatIII	SatIV		
		Cervini	Muntiacini	Capreolinae	Cervini	Muntiacini	Capreolinae	Capreolinae	Cervini	Muntiacini	Capreolinae
SatI	Cervini	88–99									
	Muntiacini	77–84	81–93								
	Capreolinae	74–81	75–83	75–86							
SatII	Cervini				86–98						
	Muntiacini				78–83	86–99					
	Capreolinae				74–85	74–78	74–88				
SatIII	Capreolinae							82–89			
SatIV	Cervini								96–99		
	Muntiacini								95–97	99	
	Capreolinae								86–97	85–94	85–95

**Table 4:** Fluorescence in situ hybridisation (FISH) patterns of the satI-IV probes in the analysed cervid species.

Species	2n	No. of Autosomes		X	SatI			SatII			SatIII			SatIV		
		Ac	Bi		Ac	Bi	X	Ac	Bi	X	Ac	Bi	X	Ac	Bi	X
**CEL**	68	64	2	Ac	+	−	+	+	+	+	−	−	−	−	−	−
DDA	68	64	2	Ac	+	−	+	+	+	+	−	−	−	−	−	−
EDA	68	64	2	Ac	+	−	+	+	+	+	−	−	−	−	−	−
CAL	66	60	4	Ac	+	−	+	+	+	+	−	−	−	−	−	−
RTI	60	48	10	Ac	+	−	+	+	+	+	−	−	−	−	−	−
REL	58	44	12	Ac	+	−	+	+	+	+	−	−	−	−	−	−
MRE	46	46	0	Ac	+	N	+	+	N	+	−	N	−	+/−	N	−
CCA	70	68	0	Bi	+/−	N	−	+	N	+	+/−	N	−	−	N	−
AAL	68	62	4	Bi	+/−	+/−	+	+	+	+	−	−	−	+/−	+/−	−
RTA	70	66	2	Bi	+	−	−	+	+	+	+/−	−	−	−	−	−
OVI	70	66	2	Bi	+	+	+	+	+	+	+/−	+/−	−	+/−	−	−

(Ac) acrocentric, (Bi) biarmed, (N) not present, (+) FISH signals on all chromosomes, (+/−) FISH signals on several chromosomes or weak signals, and (−) no FISH signals.

**Table 5:** Multiple-sequence alignment composition and selected substitution models of cervid satellite sequences.

Satellite Sequence	Number of Sequences	Alignment Length (bp)	Proportion of Gaps (%)	Substitution Model	*α*
satI	53	952	7.8	GTR + Γ	2.3
satII	51	707	6.8	K80 + Γ	2.7
satIII	7	2089	1.9	K80 + Γ	1.8
satIV	29	738	0.8	K80 + Γ	37.9

Γ—rate heterogeneity between sites modelled with the Γ distribution, and α—shape parameter of the Γ distribution.

## Supplementary Materials

The following are available online at https://www.mdpi.com/2073-4425/11/5/584/s1: **Figure S1.** CENP-B-binding motif in analysed deer species. The CENP-B-binding motif is marked in grey in the individual clones. CEL—*Cervus elaphus*, DDA—*Dama dama*, REL—*Rucervus eldii*, CAL—*Cervus albirostris*, EDA—*Elaphurus davidianus*, RTI—*Rusa timorensis,* CCA—*Capreolus capreolus*, RTA—*Rangifer tarandus*, OVI—*Odocoileus virginianus*, and MRE—*Muntiacus reevesi*. **Figure S2.** FISH patterns of the satI probe. Asterisks indicate metaphase chromosomes. **Figure S3.** FISH patterns of the satII probe. Asterisks indicate metaphase chromosomes. **Figure S4.** FISH patterns of the satIII probe. Asterisks indicate metaphase chromosomes. **Figure S5.** FISH patterns of the satIV probe. Asterisks indicate metaphase chromosomes. **Figure S6.** Karyotype of *R. eldii* showing FISH signals of probes specific to satI (green) and satII (red). **Figure S7.** FISH patterns of the satI (green) and satII (red) probes in *B. taurus* and *O. aries*. (A,B) Autologous FISH with bovine and ovine satellite probes. (C,D) Cross-hybridisation with cervid satellite probes. **Figure S8.** Bayesian phylogenetic trees constructed from cervid satellite sequences. (A) SatIII and (B) satIV. AAL—*Alces alces*, CAL—*Cervus albirostris*, CCA—*Capreolus capreolus*, CEL—*Cervus elaphus*, DDA—*Dama dama*, ECE—*Elaphodus cephalophus*, EDA—*Elaphurus davidianus*, HIN—*Hydropotes inermis*, MCR—*Muntiacus crinifrons*, MMV—*Muntiacus muntjak vaginalis*, MRE—*Muntiacus reevesi*, OHE—*Odocoileus hemionus*, OVI—*Odocoileus virginianus*, REL—*Rucervus eldii*, RTA—*Rangifer tarandus*, and RTI—*Rusa timorensis*. Circles at nodes signify nodes with posterior probability ≥ 0.95 (black) and ≥ 0.90 (grey). Unmarked nodes were not supported.
