# Supplementary material for: Sequence Analysis and FISH Mapping of Four Satellite DNA Families among Cervidae
Source: Genes (Basel). 2020 May 24;11(5):584. doi: 10.3390/genes11050584 (PMC7288315; doi:10.3390/genes11050584)
Supplement: Supplementary file 1 [file genes-11-00584-s001.zip › Supplementary_Figure_S1.pdf]

```
CEL_clone1      GGCTGGCGGGAACAGCCGGCGAGAGTGCTTGCAAAGCGGCCTTTGGAGGCAGCCGGGGAG
CEL_clone2      GGCTTATGGGAACAGCAGGCCAGAGTGCTTGCAAAGCGGCCTTTGGAGGCAGCCGGGGAG
CEL_clone3      AGCTGGCGGGAACAGCCGGCGAGAGTGCTTGCAAAGCGGCCTTTGGAGGCAGCCGGGGAG
CEL_clone4      GGCTGGCGGGAGCAGCCGGCGAGAGTGCTTGCAAAGCGGCCTTTGGAGGCAGCCGGGGAG
DDA_clone1      GCCTGCTGGGAACAGTGGGAGGGAGTGCTTGCAAACCAGCCTTTGGAGGCAGCCGGGGAG
DDA_clone2      GCCTGGCGGGAACAGCGGGCGGGAGTGCTTGCAAACCGGCCTTTCGAGGCAGACGGGGAG
DDA_clone3      AGCTGGCAGGAACAGCAGGCGAGAGTGCTTGCAAACCGGCCTTTGGAGGCAGCCGGGGAG
DDA_clone4      GCCTGGCGGGAACAGCGGCCGGGAGTG-TTGCAAACCGGCCTTTGGAGGCAGCCCGGGAG
REL_clone1      AGCTGGCGGGAACAGCCGGCGAGAGTGCTTGCAAAGCGGCCTTTGGAGGCAGATAGGGAG
REL_clone2      GGCTGGCGGGAACAGCCGGAGAGAGTGCTTGCAAAGCGGCCTTTGGAGGCAGCCGGGGAG
REL_clone3      GGCTGGCGGGAACAGCCGGCGAGAGTGCTCGCAAAGCGGCCTTTGGAGGCAGCCGGGGAG
REL_clone4      GGCTGGCGGGAACAGCCGGCGAGAGTGCTTGCAAAGCGGCCTTTGGAGGCAGCCGGGGAG
CAL_clone1      GGCTGGGGGGAACAGCCGGCGAGAGTGCTTGCAAAGCGGCCTTTGGAAGCAGCCGGGGAG
CAL_clone2      GACTGGCGGGAACAGCCGGCGAGAGTGCTTGCAAAGCGGCCTTTGGAGGCAGCCGGGGAG
CAL_clone3      GGCTGGCGGGAACAGCCGGCGAGAGTGCTCGCAAAGCGGCCTCTGGAGGCAGCCGGGGAG
CAL_clone4      GGCTGGCGGGAACAGCCGGCGAGAGTGCTTGCAAAGCGGCCTTTGGAGGCAGCCGGGGAG
EDA_clone1      GGCTGGCGGGAACAGCCGGCGAGAGTGCTTGCAAAGCGGCCTTTGGAGGCAGCCGGGGAG
EDA_clone2      GGCTGGCGGGAACAGCCAGCGAGAGTGCTTGCAAAGCGGCCTTTGGAGGCAGCCGGGGAG
EDA_clone3      GGCTGGTGGGAACAGCCGGCGAGAGTGCTTGCAAAGCGGCCTTTGGAGGCAGCCGGGGAG
EDA_clone4      GGCTGGCGGGAACAGCCGGCGAGAGTGCTTGCAAAGCGGCCTTTGGAGGCAGCCGGGGAG
RTI_clone1      AGCTGGCGGGAACAGCCGGCGAGAGTGCTTGCAAAACGGCCTTTGGAGGCAGCCGGGGAG
RTI_clone2      GGCTGGTAGGAACAGCCGGCGAGAGTGCTTGCAAAGCGGCCTTTGGAGGCAGCCGGGGAG
RTI_clone3      AGCTGGCCGGAACAGCCGGCGAGAGTGCTTGCAAAGCGGCCTTTGGAGTTAGCCGGGGAG
RTI_clone4      GGCTGGCGGGAACAGCCGGCGAGAGTGCTTGCAAAGCGGCCTTTGGAGGCAGCCGGGGAG
                  **    *** ***       ***** * ***** * **** * **   **   *****

CCA_clone1      CCGGTGAGAGTGCTTGCAAACCGGCCTTTGGAAGCAGCCGGGGAGGTCTTCCAGCGGGAG
CCA_clone2      CCGGCGAGAGTGCTTGCAAACCGGCCTTTGGAAGCAGCCGGGGAGGTCTTCCAGCGGGAG
CCA_clone3      CCGGCGAGAGTGCTTGCAAACCGGCCTTTGGAAGCAGCCGGGGAGGTCTTCCAGCGGGAG
CCA_clone4      CCGGCGAGAGTGCTTGCAAACCGGCCATTGGAAGCAGCCGGGGAGGTCTTCCAGCAGGAG
RTA_clone1      CCGGCCAGAGTGCTTGCAAACGGGGATTTGCACGCAGGCGGGGAGGTCTTCCAGAGGGAG
RTA_clone2      CCGGCCAGAGTGCTTGCAAACGGGGATTTGCACGCAGGCGGGGAGGTCTTCCAGAGGGAG
RTA_clone3      CCGGCCAGAGTGCTTCCAAACCAGC-CTTGGAAGGAGCCGGGGAGGTCTTCGAGCGGGAG
RTA_clone4      CCGGCCAGAGTGCTTGCAAACGGGGATTTGCACGCAGGCGGGGAGGTCTTCCAGAGGGAG
OVI_clone1      CCGGCCAGAGTGCTTCCCAAACGGCCTTTGGAGGCAGGTGGGGAGGTCTGCCGGAGGGAG
OVI_clone2      CCGGCCAGAGTGCTTCCCAAACGGCCTTTGGAGGCAGGCGGGGAGGTCTGCCGGAGAGAG
OVI_clone3      CCGGCCAGAGTGCTTCCAAAACGGCCTTTGGAGGCAGGCGGGGAGGTCTGCCAGAGGGAG
OVI_clone4      CCGGCCAGAGTGCTTCCCAAACGGCCTTTGGAGGCAGGCGGGGAGGTCTGCCCGAGGGAG
                ****  ********* * **   *   *** * * **  ********** *  *   ***

MRE_clone1      GGAGCAGTGTGCTCAGCTTGCGGGAATCGCGGGCCTCCCTCGGGGAGAGTGCTTGCAAAC
MRE_clone2      GGAGCAGGGTGCTCAGCTTGCGGGAATCGCGGGCCTCCCTCGGGGAGAGTGCTTGCAAAC
MRE_clone3      GAAGCAGAGTGCGCGGCTTGCGGGAAACGTGGGCTTTCCTCGGGTATAGTGCTTGCAAAC
MRE_clone4      GGAGCAGTGTGCTCAGCTTGCGGGAATCGCGGGCCTCCCTCGGGGAGAGTGCTTGCAAAC
                * ***** **** * *********** ** **** * ******* * **************
```
