# Supplementary figures and images for: Sequence Analysis and FISH Mapping of Four Satellite DNA Families among Cervidae
Source: Genes (Basel). 2020 May 24;11(5):584. doi: 10.3390/genes11050584 (PMC7288315; doi:10.3390/genes11050584)

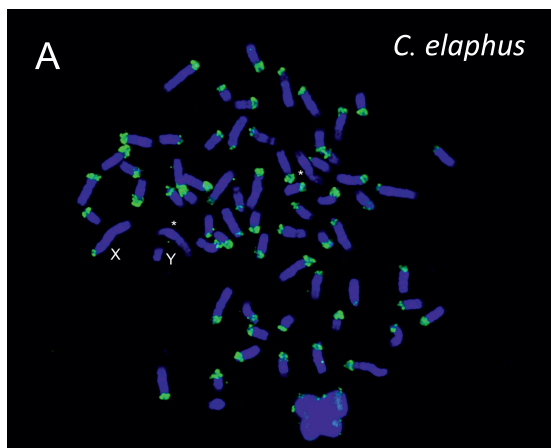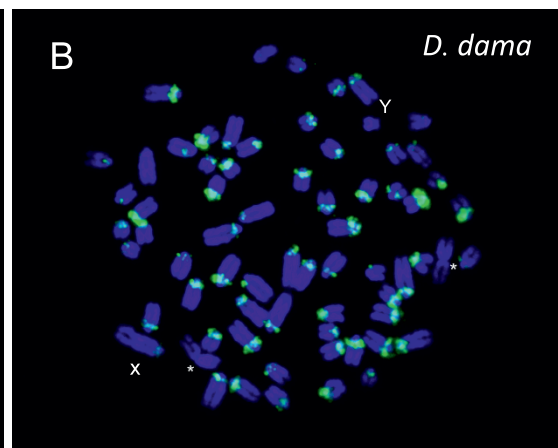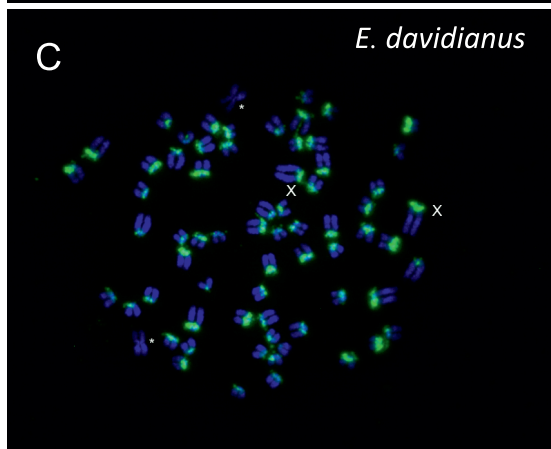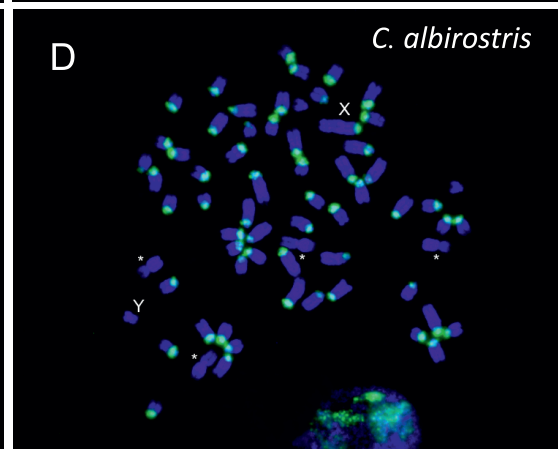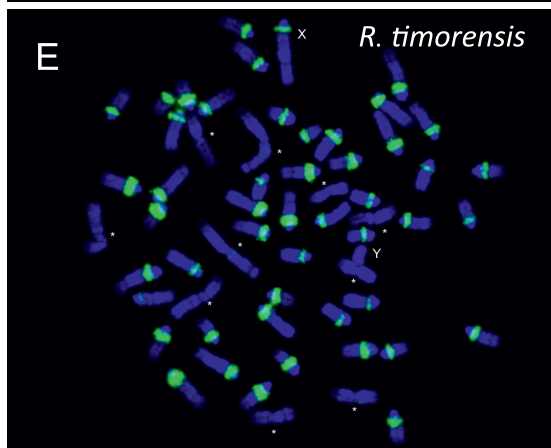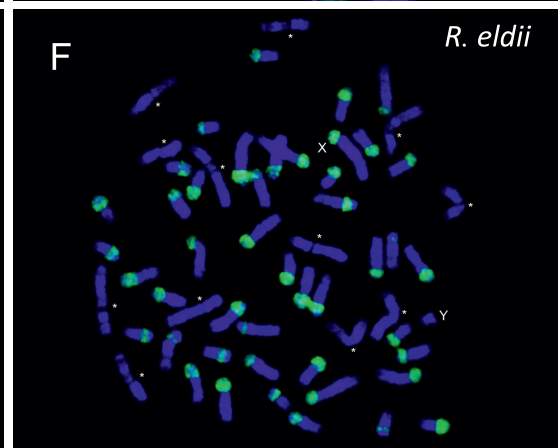

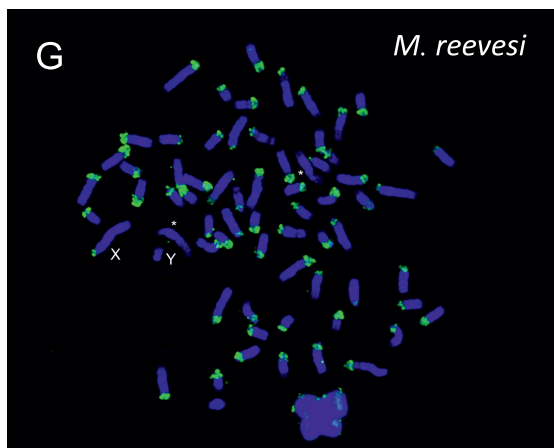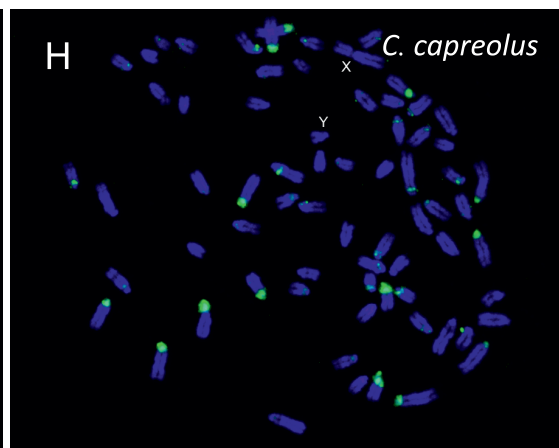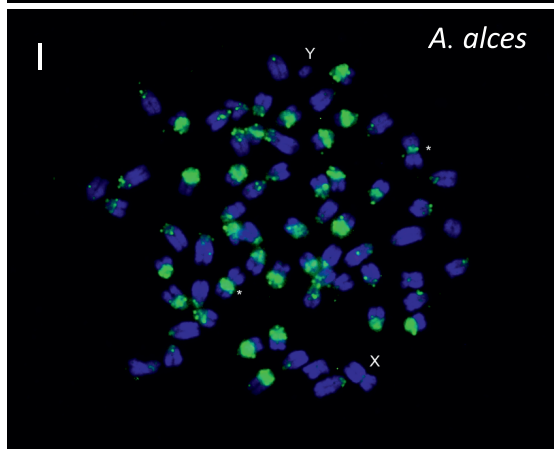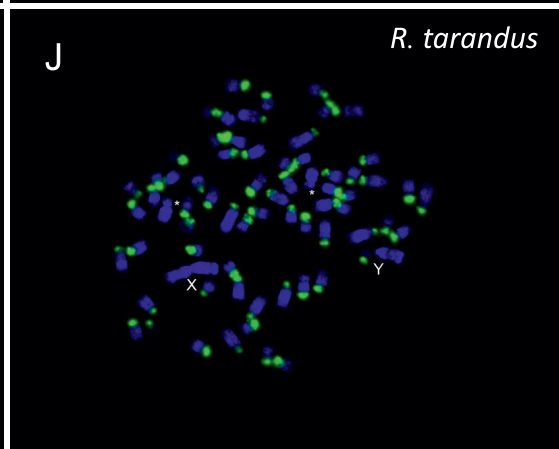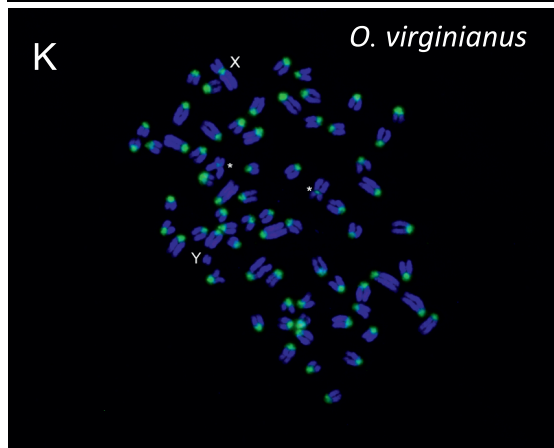

Supplement: Supplementary file 1 [file genes-11-00584-s001.zip › Supplementary_Figure_S2.pdf]

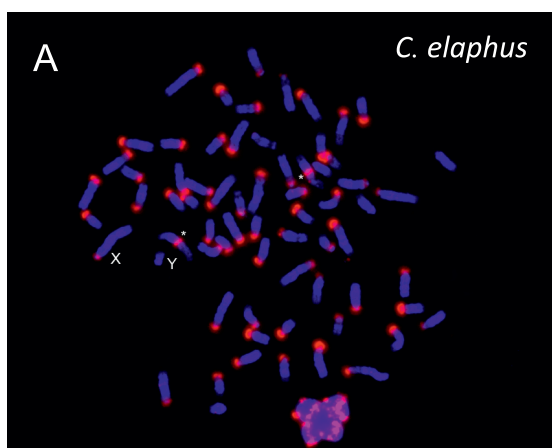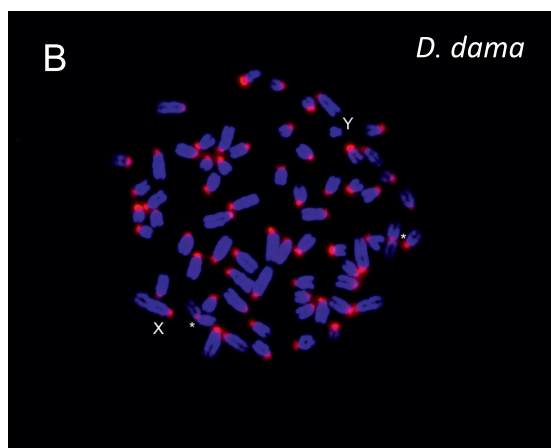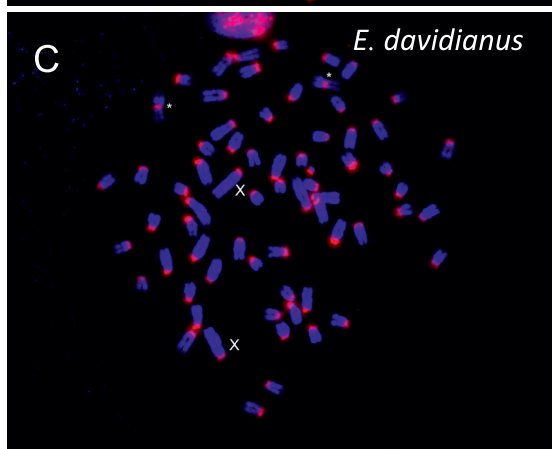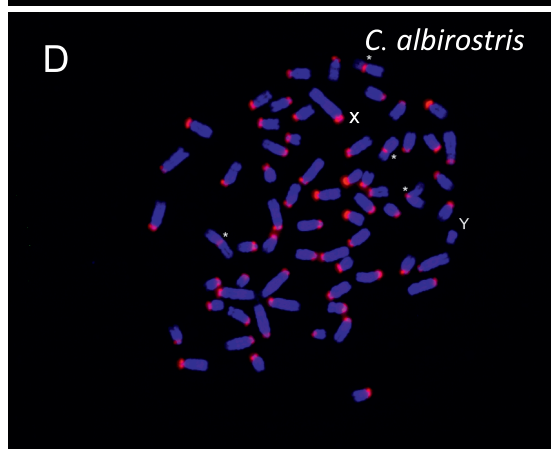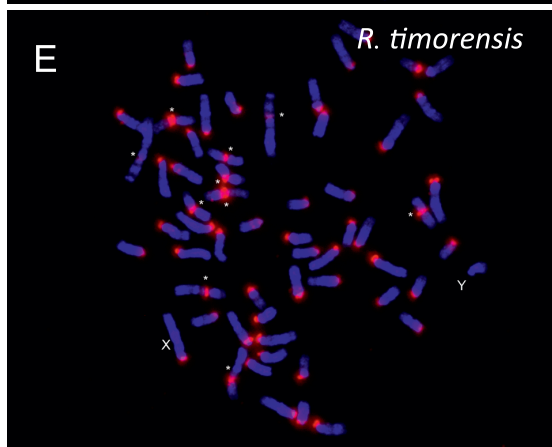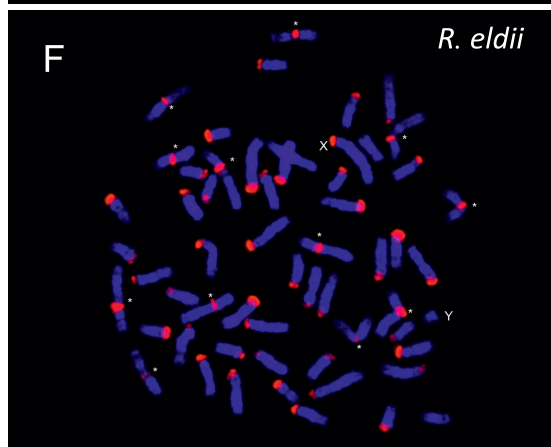

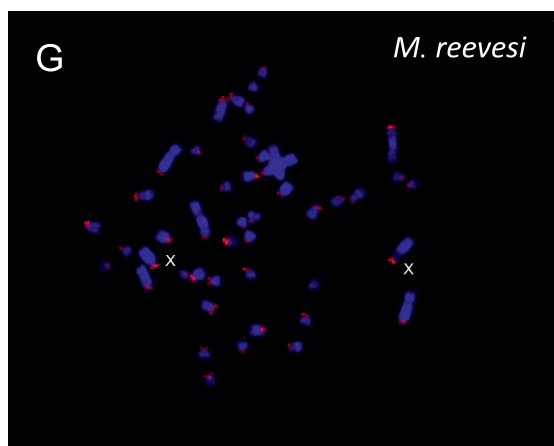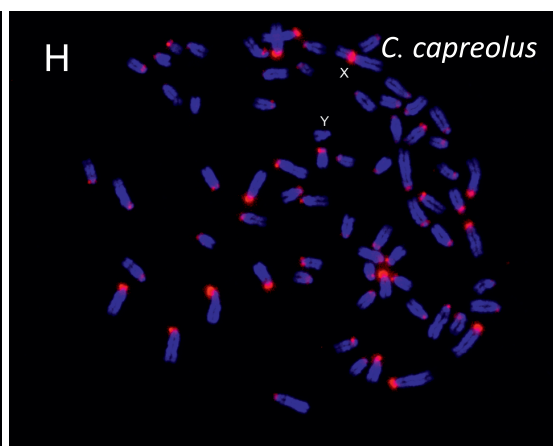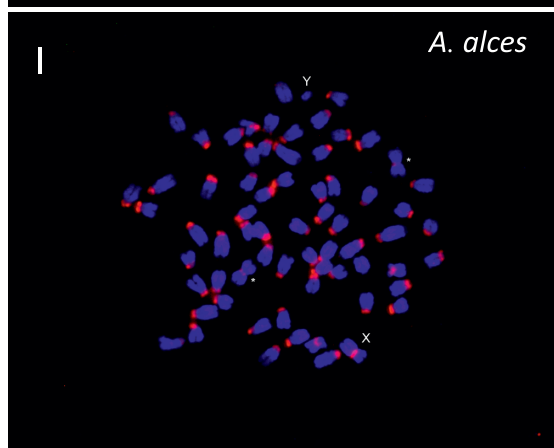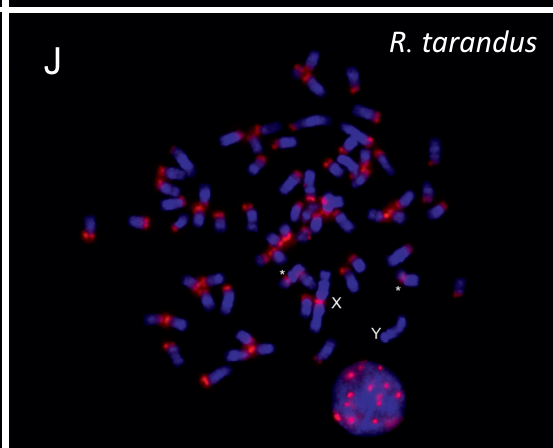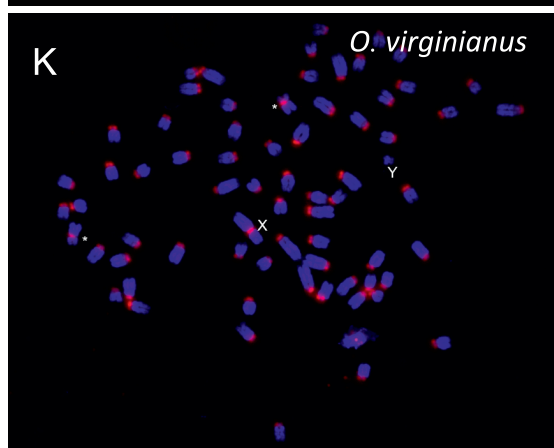

Supplement: Supplementary file 1 [file genes-11-00584-s001.zip › Supplementary_Figure_S3.pdf]

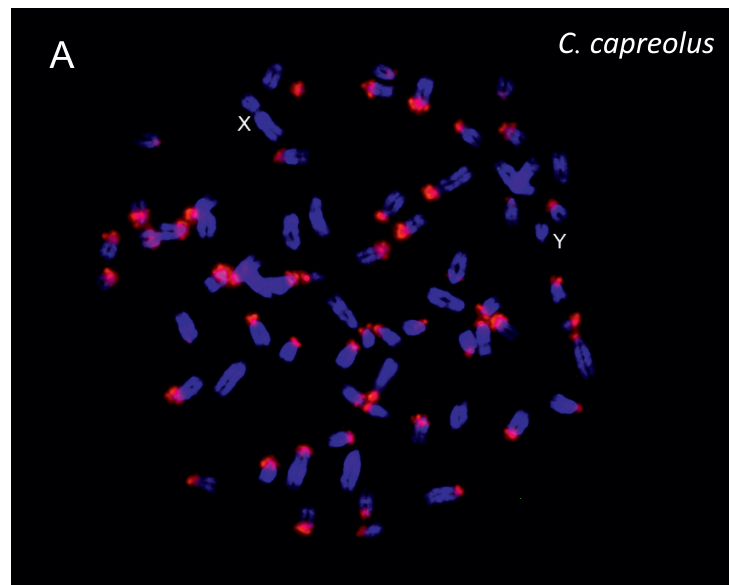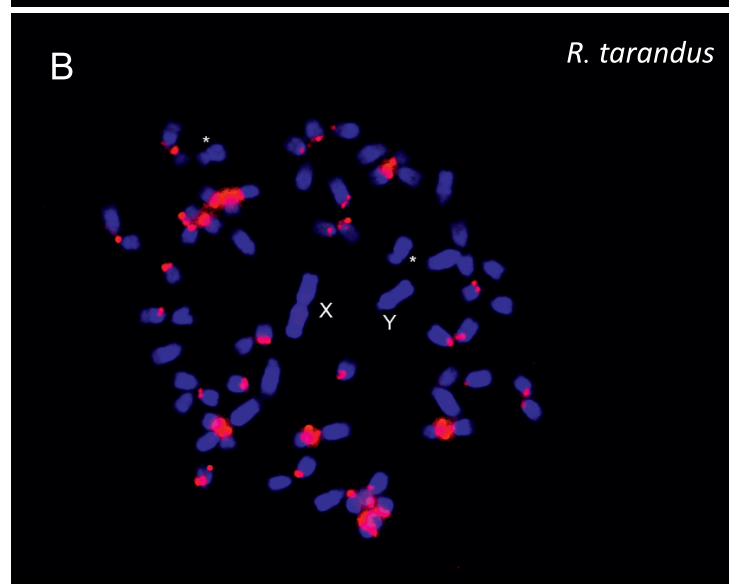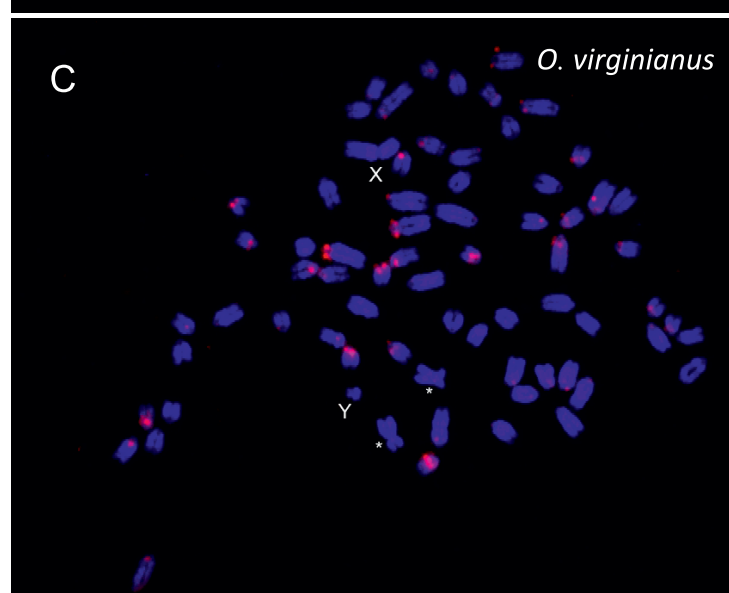

Supplement: Supplementary file 1 [file genes-11-00584-s001.zip › Supplementary_Figure_S4.pdf]

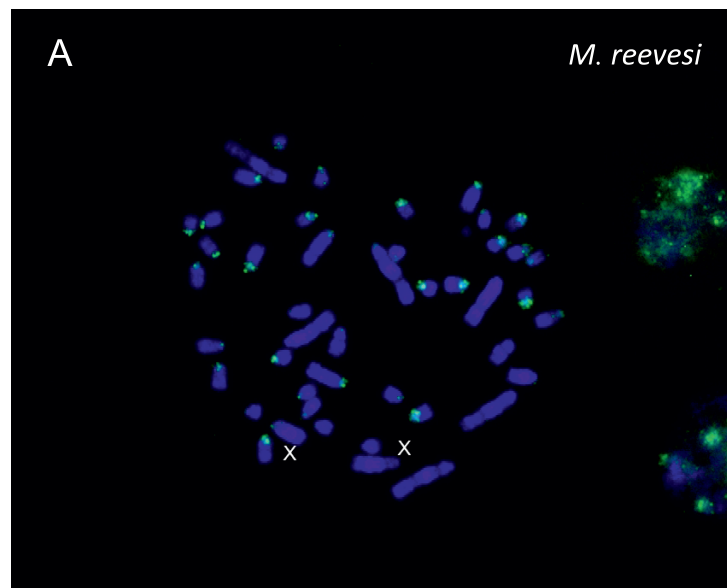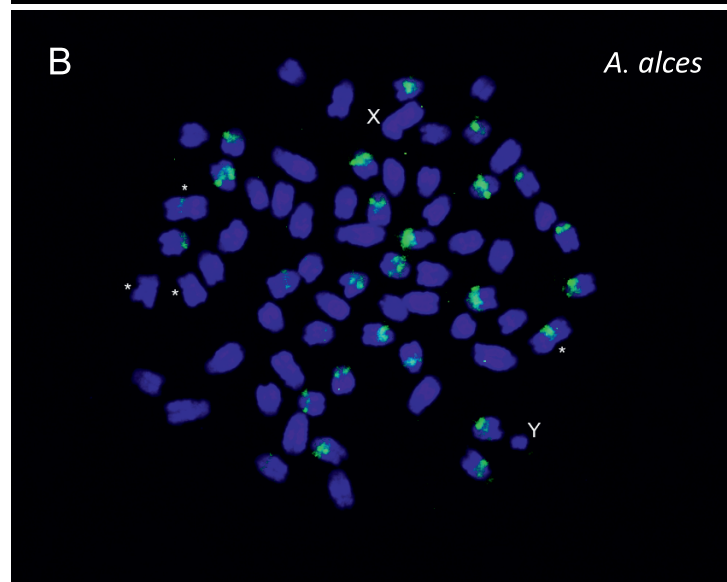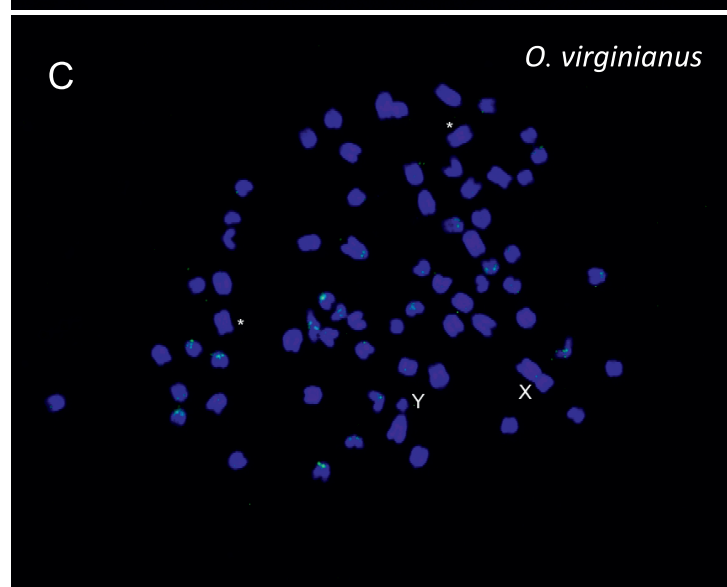

Supplement: Supplementary file 1 [file genes-11-00584-s001.zip › Supplementary_Figure_S5.pdf]

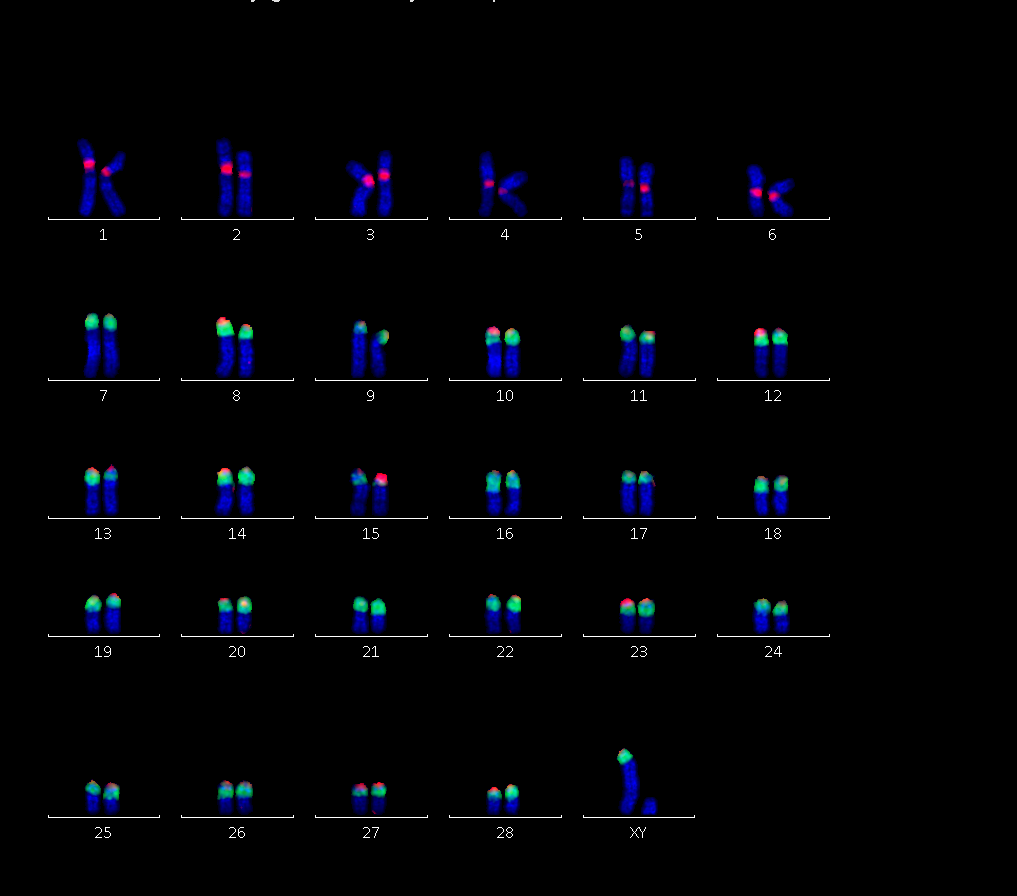

Supplement: Supplementary file 1 [file genes-11-00584-s001.zip › Supplementary_Figure_S6.TIF]

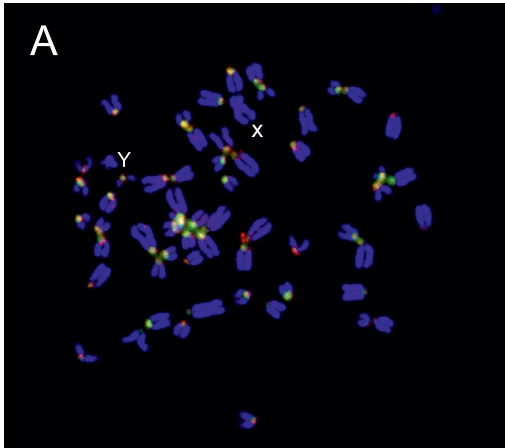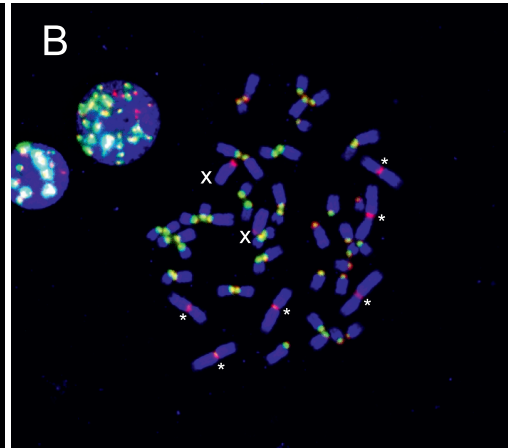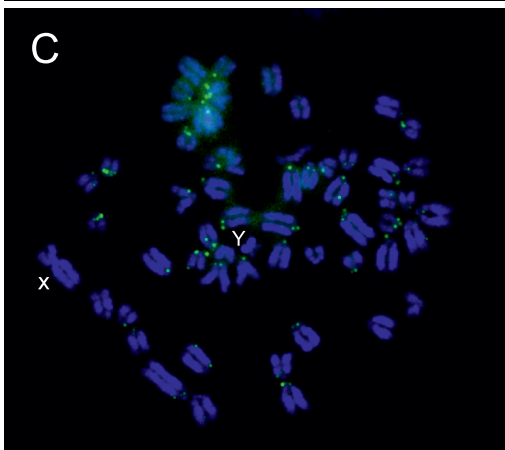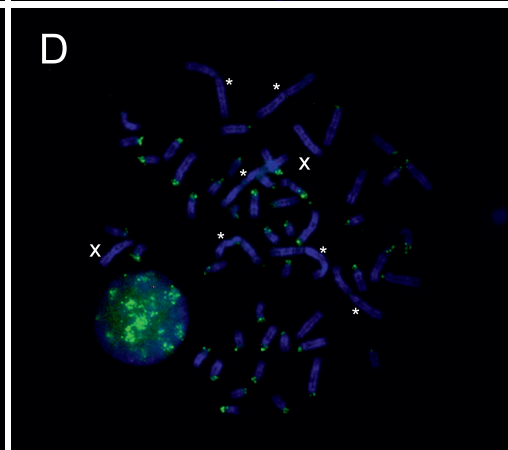

Supplement: Supplementary file 1 [file genes-11-00584-s001.zip › Supplementary_Figure_S7.pdf]

**A**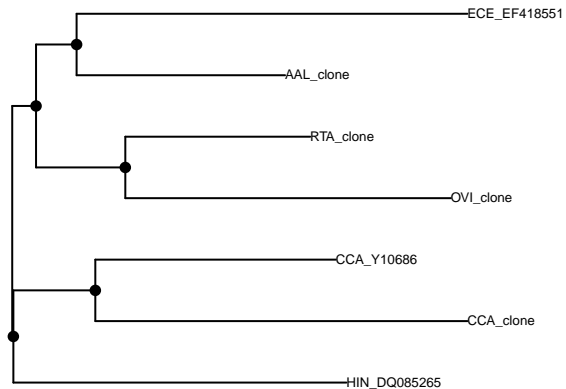

0.05

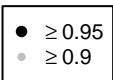**B**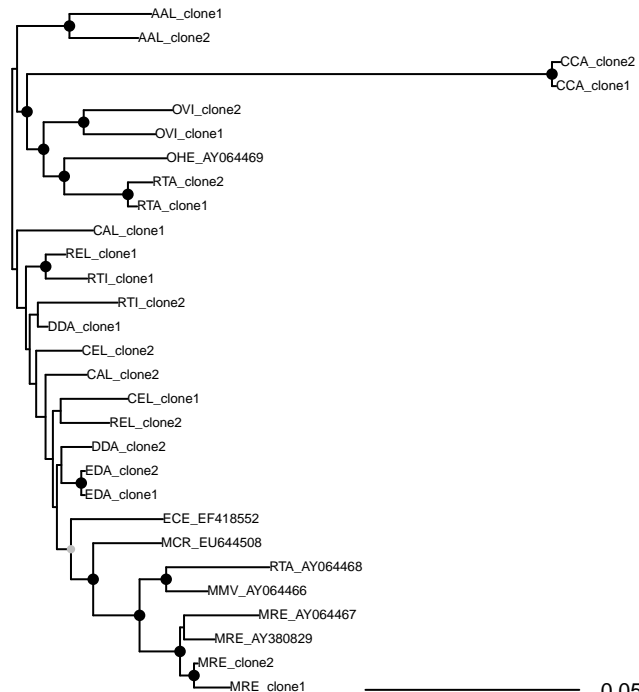

0.05

Supplement: Supplementary file 1 [file genes-11-00584-s001.zip › Supplementary_Figure_S8.pdf]
